# Quality Assessment of PBM Protocols for Oral Complications in Head and Neck Cancer Patients: Part 1

**DOI:** 10.3389/froh.2022.945718

**Published:** 2022-07-07

**Authors:** Margherita Gobbo, Elisabetta Merigo, Praveen R. Arany, René-Jean Bensadoun, Alan Roger Santos-Silva, Luiz Alcino Gueiros, Giulia Ottaviani

**Affiliations:** ^1^Unit of Oral and Maxillofacial Surgery, Ca' Foncello Hospital, Treviso, Italy; ^2^Faculty of Dentistry, Côte d'Azur University, Nice, France; ^3^Oral Biology, Surgery and Biomedical Engineering, University at Buffalo, Getzville, NY, United States; ^4^Department of Radiation Oncology, Centre de Haute Energie, Nice, France; ^5^Oral Diagnosis Department, Piracicaba Dental School, University of Campinas, São Paulo, Brazil; ^6^Department of Clinic and Preventive Dentistry, Federal University of Pernambuco, Recife, Brazil; ^7^Department of Surgical, Medical and Health Sciences, University of Trieste, Trieste, Italy

**Keywords:** oral cancer, photobiomodulation, oral mucositis, dysgeusia, xerostomia, dermatitis, trismus, oedema

## Abstract

**Background:**

Radiotherapy and chemotherapy are frequently employed in head and neck cancer (HNC) patients causing significant side effects that impair life quality and prognosis. Photobiomodulation (PBM) has become a growing approach to managing such oral complications. Despite its proven efficacy and absence of contraindications, there is still a lack of universally accepted disease-specific PBM protocols.

**Objective:**

A narrative review was conducted to identify the current proposals relating to the use of PBM to treat complications of oncological treatments in HNC patients.

**Methods:**

An electronic search in PubMed and Scopus databases was performed with the following keywords: (“photobiomodulation” OR “PBM” OR “laser therapy” OR “LLLT” OR “laser”) AND (“head and neck cancer” OR “oral cancer”) AND (“mucositis” OR “oral mucositis” OR “dysgeusia” OR “oedema” OR “xerostomia” OR “dermatitis” OR “trismus”) until October 2021.

**Results:**

A total of 35 papers were included in the narrative review. Oral mucositis was the most studied complication, and advisable protocols are conceivable. Although there is a growing interest in PBM to manage of xerostomia, radiodermatitis, pain, and trismus, literature is still scarce to propose a universally feasible protocol.

**Conclusions:**

PBM therapy could significantly prevent or reduce the severity of many side effects related to cancer therapies. More research is needed to obtain recommendations over the preferable parameters.

## Introduction

Head and neck cancer (HNC) is primarily treated with surgery in combination with radiotherapy (RT) and/or chemotherapy (CT). RT and/or CT in the head and neck region (HNR) have several side effects that can be debilitating and heavily affect patients' quality of life (QoL) and prognosis. The most common side effects include oral mucositis (OM), xerostomia, dysgeusia, oedema, radiation caries, radiodermatitis, and trismus [1]. These spectra of ailments share a common etiopathology of these complications involving sensitization and tissue damage by the oncotherapy agent. Photobiomodulation (PBM) is a non-invasive light therapy increasingly being applied in supportive care for cancer patients. Its main properties cover the field of wound healing and inflammation. However, there is still no clear consensus over the standard protocols and devices to employ. Recent insights have been made about molecular mechanisms, biological responses, and biomarkers for safe and effective PBM treatments [2, 3]. Concurrently, there have been significant advancements with device technologies, increasing availability of wavelengths, and precise control of the beam and output parameters [4]. Therefore, the objective of the present paper was to produce a narrative review of the available scientific evidence to identify the current proposals and related protocols of PBM to manage the most prevalent complications of oncological treatments in the HNR.

## Methods

An electronic search in the PubMed and Scopus databases was conducted with the following keywords: (“photobiomodulation” OR “PBM” OR “laser therapy” OR “LLLT” OR “laser”) AND (“head and neck cancer” OR “oral cancer”) AND (“mucositis” OR “oral mucositis” OR “dysgeusia” OR “oedema” OR “xerostomia” OR “dermatitis” OR trismus) until October 2021. Papers in languages different from English, Italian, Spanish, Portuguese, and French were excluded. Only original articles and reviews were initially included, excluding short reports and case reports. Further, articles not specifying laser protocol were also excluded. A global group of experts in oral medicine, oncology, radiation biology, and PBM examined and discussed this literature to further develop consensus.

## Results

A total of 148 studies were obtained after the electronic search. Two different reviewers read all abstracts. After the abstract screening, 58 were excluded, and 90 were subdivided among reviewers' full-text analyses performed independently by two reviewers. After the full-text screening, 35 papers were included in the narrative review. The majority of papers were about preventing or treating more than one side-effect. Twenty-seven studies dealt with OM, 10 with xerostomia, 4 with radiodermatitis, and 2 with pain and trismus. Other interesting topics included the evaluation of QoL outcomes, systemic analgesia, functional impairment, nutritional status, survival, interruption of RT, adherence, cost-effectiveness, safety, feasibility, and tolerability of PBM. In general, no adverse effects were reported, and all authors supported safety and tolerability. Although clinical time constraints and patient compliance were often considered limitations to PBM therapy, feasibility was high. Further detailed analysis of these results will be conducted in another review by our group. In the phase of full-text screening, reviews and systematic reviews were excluded as they did not mention detailed laser parameters.

### Study Characteristics

Overall, 7 papers were published between 1999 and 2010, 19 papers between 2011 and 2019, and 9 papers in the last 2 years, witnessing the increasing interest in the field of PBM applied to supportive care in cancer patients (Table 1). A total of 14 studies investigated the role of PBM in preventing the onset of the side effect, 13 in treating the complications, and 8 studies mentioned both protocols. Twenty-two studies included HNC patients subjected to RT sessions alone or combined with surgery, whereas 13 studies included HNC patients subdued to combined CT and RT, with exclusive regimens or as adjuvants to surgical treatments.

**Table 1:** Characteristics of studies included in the narrative review.

**References**	**Sample size**	**Type of study**	**Cancer treatment**	**Topics**	**Synthesis of main results**
Bensadoun et al. [5]	PBM group: 15 patients Placebo group: 15 patients Mean age: 60.4 (36–78) years	Multi-center double blind randomized controlled trial Preventive PBM	CT/RT	Oral mucositis Nutritional status	PBM therapy reduced severity and duration of OM associated with RT. In addition, there is a tremendous potential for using PBM in combined treatment protocols utilizing concomitant CT and RT
Arun Maiya et al. [6]	PBM group: 25 patients, 54 ± 1 years Control group: 25 patients, 53 ± 1 years Gender ratio M:F = 2:1	Prospective randomized blind controlled study Preventive and therapeutic PBM	RT	Oral mucositis	PBM delayed the time of onset, attenuated the peak severity and shortened the duration of OM and pain, controls had more feeding tubes
Lopes et al. [7]	PBM group: 25M, 6F Placebo group: 25M, 4F Mean age: 57.4 ± 13.9 (28–88) years	Randomized clinical trial Preventive PBM	RT	Oral mucositis Xerostomia	The group of patients submitted to RT and PBM had lower incidence of xerostomia, OM and pain when compared to the group treated with RT without PBM
Arora et al. [8]	PBM group: 11 patients Control group: 13 patients Age range: 55–59 years Gender ratio M:F = 1:1	Single-center, prospective, controlled study Preventive PBM	RT	Oral mucositis Systemic analgesia Functional impairment	PBM applied prophylactically during RT can reduce the severity of OM, the severity of pain, and the functional impairment
Simões et al. [9]	39 patients divided in 3 groups Ages range: 15–79 years	Prospective non-controlled study Therapeutic PBM	RT	Oral mucositis	PBM 3×/week was better than one and the combination of low power laser with high power laser is more effective for pain relief but prolongs healing time. For improving the patient's QoL, the most significant effect is the control of pain observed when high power laser was used
Zanin et al. [10]	PBM group: 31M, 5F Control group: 29M, 7F Age range: 34–80 years	Randomized, double-blinded, placebo-controlled clinical trial Preventive and therapeutic PBM	CT/RT	Oral mucositis Quality of life	A 660-nm diode laser was effective in the prevention and treatment of OM in patients undergoing RT and CT, providing them more comfort and a better QoL
Lima et al. [11]	PBM group: 12 patients AH: 13 patients Mean age: 55.82 (33–80) years Male 90.91%, female 9.08%	PBM vs. aluminum hydroxide Preventive PBM	CT/RT	Oral mucositis Quality of life	The prophylactic use of both treatments seems to reduce the incidence of severe OM lesions. However, the PBM was more effective in delaying the appearance of severe OM
Carvalho et al. [12]	PBM group: 25M, 10F Mean age: 56.2 ± 14.5 (22–94) years Control group: 21M, 14F Mean age: 58.1 ± 10.9 (35–79) years	Double blind randomized controlled study Preventive and therapeutic PBM	CT/RT	Oral mucositis	PBM appears to present promising results, both in controlling OM intensity and pain-related
Oton-Leite et al. [13]	PBM group: 22M, 8F Placebo group: 27M, 3F Median age: 55.6 (30–80) years	Therapeutic PBM	RT	Oral mucositis Quality of life	PBM improves OM and consequently the QoL of patients with head and neck cancer undergoing RT and justifies the adoption of PBM in association with conventional cancer treatment
Gautam et al. [14]	PBM group: 97M (87.4%), 14F (12.6%) Mean age: 55.18 ± 11.70 years Placebo group: 92M (83.6%), 18F (16.4%) Mean age: 55.95 ± 11.61 years	Prospective, single centered, triple blinded, randomized controlled trial Preventive PBM	CT/RT	Xerostomia Quality of life Systemic analgesia and functional impairment	Preventive PBM decreased the incidence of CT/RT severe OM and pain, dysphagia and opioid analgesics use and unplanned treatment interruption. It can be considered as non-traumatic modality for the treatment of OM and its associated morbidity
^*^Gouvêa de Lima et al. [15]	PBM: 27M, 10F Mean age: 53.1 ± 9.4 years Placebo: 30M, 8F Mean age: 53.2 ± 10.3 years	Phase III, randomized, double-blind study Preventive PBM	CT/RT	Xerostomia Systemic analgesia and functional impairment RT interruption	PBM did not improve pain control and it was not effective in reducing grade 3 and 4 OM, although a marginal benefit could not be excluded. It reduced RT interruptions in HNC patients, which might translate into improved CRT efficacy
Gautam et al. [16]	PBM group: 50M (91%), 5F (9%) Mean age: 51.71 ± 11.94 years Placebo group: 48M (87%), 7F (13%) Mean age: 52.60 ± 12.51 years	Prospective, unicentric, double blinded, randomized controlled trial Preventive and therapeutic PBM	CT/RT	Oral mucositis Nutritional status Systemic analgesia and functional impairment	PBM showed better treatment outcomes in preventing and treating the CT/RT induced severe OM than placebo in HNC patients. Incidence of severe oral pain, opioid analgesics use and total parenteral nutrition was less in laser than placebo patients. Hence, it can be considered as a therapeutic modality for improving OM associated decreased oral functions and QoL in these patients
Oton-Leite et al. [17]	PBM group: 30 patients Control group: 30 patients Male: 81.6% Mean age: 56.1 ± 12.4 (30–81) years	Prospective randomized controlled trial Preventive and therapeutic PBM	RT	Oral mucositis Xerostomia	Greater pain scores and lower salivary flows (stimulated and unstimulated) were observed in the follow-up periods in the control group. Better outcomes were observed in the PBM group indicating lower degrees of OM, pain and higher salivary flow (*p* < 0.05)
Antunes et al. [18]	PBM group: 42M, 5F Mean age: 53.5 ± 6.9 years Control group: 40M, 7F Mean age: 55.7 ± 8.6 years	Prospective, randomized, double-blind, placebo-controlled phase III trial Preventive PBM	CT/RT	Oral mucositis	PBM is effective in preventing CT/RT-induced grades 3–4 OM in HNC patients
Gautam et al. [19]	PBM group: 97M (88%); 13F (12%) Mean age: 55 ± 11.52 years Control group: 92M (84%); 18F (16%) Mean age: 56 ± 11.80 years	PBM vs. placebo Therapeutic PBM	CT/RT	Oral mucositis Quality of life	PBM was effective in improving the patient's subjective experience of OM and QoL in HNC patients receiving CT/RT
Gobbo et al. [20]	PBM group: 29M, 13F Control group: 14M, 7F Mean age: 65.4 ± 10.3 (43–89) years	Case-control retrospective Therapeutic PBM	RT	Oral mucositis Nutritional status	PBM has to be considered as a powerful weapon in practitioners' hands and should become part of everyday practice and strategy for oncological patients
Oton-Leite et al. [21]	PBM group: 9M, 3F Control group: 12M, 1F	Original study Therapeutic PBM	CT/RT	Oral mucositis Xerostomia Salivary mediators	PBM brought a clinical improvement in OM in HNC patients undergoing CT/RT. This resulted in the attenuation of the inflammatory process and less required repair
Gautam et al. [22]	PBM group: 22 patients Mean age: 71.57 ± 7.27 years Placebo group: 24 patients Mean age: 69.67 ± 8.68 years	A randomized, double blinded, placebo-controlled trial Therapeutic PBM	RT	Oral mucositis Nutritional status Systemic analgesia and functional impairment	PBM was effective in reducing the severity and duration of RT induced OM and oral pain in elderly HNC patients. Also need for opioid analgesics, total parenteral nutrition and radiation break was less in laser treated patients. PBM can be considered a therapeutic modality against RT-induced OM in elderly HNC patients
Gonnelli et al. [23]	PBM group: 15M, 2F Mean age: 56.6 (35–74) years Control group: 9M, 1F Mean age: 58.5 (51–68) years	Prospective randomized study Therapeutic PBM	RT	Xerostomia	PBM seems to be an efficient tool for mitigation of salivary hypofunction in patients undergoing RT for HNC
Palma et al. [24]	PBM group: 21M, 8F Mean age: 61 (48–74) years	Prospective non-controlled study Therapeutic PBM	RT	Xerostomia	PBM seems to be effective to mitigate salivary hypofunction and increase salivary pH of patients submitted to RT for HNC treatment. As a final result, an evident improvement in QoL could be achieved
Elgohary et al. [25]	Group A (LIUS and TET): 11M, 9F; 61.00 ± 6.16 years Group B (LLLT and TET): 10M, 10F; 60.75 ± 5.09 years Group C (TET): 12M, 8F; 62.85 ± 5.77 years	Original study Traditional Exercise Therapy (TET) vs. LLLT and Low Intensity UltraSound (LIUS) Therapeutic PBM	RT	Pain and trismus Quality of life	All the three approaches were beneficial in managing TMJ dysfunctions. LIUS has a more superior effect when combined with the TET program in comparison to LLLT when combined with the same types of exercises in the treatment of trismus and its related pain among patients with HNC
González-Arriagada et al. [26]	PBM group: 87M, 21F Control group: 86M, 22F	Case-control study Therapeutic PBM	RT	Oral mucositis Xerostomia Pain and trismus Dermatitis RT interruption	PBM and the inclusion of oral care professionals in the multidisciplinary oncologic team contribute to reducing the morbidity resulting from OM and other collateral effects and would increase the QoL of RT HNC patients
Guedes et al. [27]	PBM group: 58 patients (88% M, 12% F) Median age: 59.5 (30–85) years	Prospective cohort study Therapeutic PBM	RT	Oral mucositis Survival/recurrence	PBM with high doses of laser energy produces a small improvement in the prevention of RT-induced OM and did not significantly increase the risk of neoplastic recurrence
Legouté et al. [28]	PBM group: 37M, 5F Mean age: 58 (53–62) years Placebo group: 38M, 3F Mean age: 58 (53–68) years	Prospective randomized study Preventive PBM	CT/RT	Oral mucositis Systemic analgesia and functional impairment Safety	PBM was well-tolerated with a good safety profile, which promotes its use in clinical routine for severe OM treatment
Rezk-Allah et al. [29]	PBM group: 80 patients Median age: 55.2 years	Original study Therapeutic PBM	CT/RT	Oral mucositis Cytokines	PBM is well-tolerated and improves OM. It may be useful to improve the symptoms of CT-induced OM
Bourbonne et al. [30]	PBM group: 31M, 9F Median age: 61 (45–76) years	Prospective not controlled study Therapeutic PBM	RT	Oral mucositis RT interruption	The surface laser applied transcutaneously seems to allow patients to tolerate treatment without interruption and to develop low mucosal toxicity rates
Morais et al. [31]	PBM group: 49M (80.3%); 22F (19.7%) Mean age: 58.6 ± 9.9 years	Original Prospective study Preventive PBM	RT	Oral mucositis Xerostomia Quality of life Survival RT interruption	The PBM associated with a rigorous and well-controlled preventive oral care protocol resulted in satisfactory control of oral adverse effects, reduction of QoL impacts, and interruption of RT regimen due to severe OM
^*^Dantas et al. [32]	PBM group: 23M, 7F Mean age: 55.9 ± 11.1 years Control group: 24M, 2F Mean age: 57.9 ± 9.5 years	Case control prospective study Preventive PBM	CT/RT	Oral mucositis Xerostomia	PBM was not effective for the prevention of OM, salivary stimulation, or pain management in oral cavity cancer patients undergoing CT/RT of the head and neck region
Park et al. [33]	PBM group: 42 patients Mean age: 55.61 ± 9.84 (19–79) years	Prospective, pilot study Preventive PBM	RT	Dermatitis Safety	PBM is safe and feasible. It might be effective to reduce the severity of acute RD in patients receiving 60 Gy or higher dose of RT in the head and neck area
De Carvalho et al. [34]	PBM group: 56M, 17F Mean age: 55.8 ± 11.9 (29–79) years	Double-blind, randomized prospective study Preventive and therapeutic PBM	RT	Oral mucositis	PBM protocol used in group 1 (660 nm, 15 mW, 3.8 J/cm^2^) presented better ability to delay grade II OM and lower pain scores. The protocol used in group 2 presented similar results to group 3 for the management of RT-induced OM
^*^Ribeiro et al. [35]	PBM group: 14M, 6F Mean age: 64 ± 10.3 years	Analytical cross-sectional Preventive PBM	RT	Xerostomia	The use of PBM did not prevent the reduction of salivary flow associated with RT, but it did appear to prevent patients from progressing to higher degrees
de Pauli Paglioni et al. [36]	PBM group: 107M (73.8%), 38F (26.2%) Mean age: 58.9 ± 10.19 years	Retrospective, cohort study Preventive PBM	RT	Oral mucositis Nutritional status	PBMT may offer the potential to reduce the occurrence and severity of OM and associated pain and reducing the use of enteral feeding and opioid analgesic use
Martins et al. [37]	PBM group: 20M, 5F Mean age: 60.32 ± 9.76 years Control group: 21M, 2F Mean age: 59.13 ± 13.68 years	Double-blind randomized controlled trial Preventive and therapeutic PBM	RT	Oral mucositis	PBMT is effective in the prevention and treatment of severe OM
Robijns et al. [38]	PBM group: 23M, 5F Mean age: 64.06 ± 11.78 years Placebo group: 16M, 2F Mean age: 65.06 ± 10.37 years	Randomized, placebo-controlled trial Preventive PBM	RT	Dermatitis	PBM significantly reduces the severity of RD and improves the patients' QoL during their RT course
Bensadoun et al. [39]	72 patients (A1: 17M, 5F; A2: 8M, 1F; A3: 23F; A4: 18F) Median age: 61.4 years	Multicentric, prospective, non-comparative study Preventive and therapeutic PBM	RT	Oral mucositis Dermatitis Safety	CareMin650 is feasible, safe, and well-tolerated for preventive or curative treatment of OM and RD in cancer patients treated with RT. Preliminary efficacy results are promising

*Topics in black color: theme discussed in the present review, topics in gray color: theme not considered in the present review. M, male; F, female; PBM, photobiomodulation; RT, radiotherapy; CT, chemotherapy; OM, oral mucositis; QoL, quality of life; HNC, head and neck cancer; TET, traditional exercise therapy; LLLT, low level laser therapy; LIUS, low intensity ultrasound; TMJ, temporomandibular joint; RD, radiodermatitis*.

* *Lack of reported benefits after PBM therapy*.

### Light Parameters

Detailed characteristics of PBM protocols in included studies are outlined in Table 2. We noted considerable variations in the types of used lasers, mode of application, frequency of treatment, and treatment parameters. Our analysis precludes robust clinical guidelines. Nonetheless, an overview of the most relevant protocols for each category is outlined to assist clinical implementation.

**Table 2:** Laser parameters of the studies included in the narrative review.

**References**	**Type** **brand**	**Wavelength**	**Mode (CW/Pulse)**	**Format (fiber, array)**	**Contact or distance**	**Power output (mW)**	**Irradiance (mW/cm^**2**^)**	**Spots/area**	**Time/site**	**Time/session**	**Repetitions**	**Fluence/site**	**Fluence/session**	**Total fluence**
Bensadoun et al. [5]	Low-energy He-Ne laser (Fradama Geneva, Switzerland)	632.8 nm	CW	Fiber	0.5 mm	60 mW	NS	1 cm^2^/point 9 points	33 s per spot (Nice and Marseilles) 80 s per spot (Reims)	5 min/session (Nice and Marseilles) 12 min/session (Reims)	5 days/week (Monday to Friday) for 7 consecutive weeks	2 J/cm^2^	18 J	3 J/cm^2^
Arun Maiya et al. [6]	He-Ne laser (Electro care Ltd. Laser 2001, India)	632.8 nm	NS	Fiber	NS	10 mW	NS	NS	NS	3 min/session	5 days/week	1.8 J/cm^2^	NS	NS
Lopes et al. [7]	InGaAlP laser	685 nm	NS	Fiber	Contact	50 mW (nominal power) 35 mW (real power)	Diameter of 400 μm	0.028 cm^2^ 19 points	ns	58 s	10 days	2 J/point	NS	70 J/cm^2^
Arora et al. [8]	He-Ne laser (Electro Care Ltd, Laser 2001, Chennai, India)	632.8 nm	Pulse (10 Hz) for 8 days, then CW for 25 days	Scanner for 8 days, fiber for the following 25 days	Distance	10 mW	NS	NS	5 min/site on 6 sites	First 8 days: 5 mins supine position, following 25 days: 30 min	33 sessions	1.8 J/cm^2^	NS	NS
Simões et al. [9]	Low Power Laser: InGaAlP diode laser (Twin Flex III Evolution, MMOptics® Ltda, São Carlos, Brazil) Combined Low/High Power Lasers: GaAlAs diode laser (Soft Lase, Zap Laser Ltd, Pleasant Hill, CA)	Low Power Laser: 660 nm Combined Low/High Power Lasers: 808 nm	CW	Fiber	Non-contact 1 cm from the lesion	40 mW	Low Power Laser: 40 mW/cm^2^ Combined Low/High Power Lasers: 1 W/cm^2^	0.036 cm2	Low Power Laser: 6 s per 62 points Combined Low/High Power Lasers: 10 s on ulcers	Low Power Laser: 372 s Combined Low/High Power Lasers: ns	1–3 times/week for 8 months	Low Power Laser: 0.24 J/point	Low Power Laser: 6 J/cm^2^	Low Power Laser: 3.8 J/cm^2^
Zanin et al. [10]	AlGaInP diode laser (Bio Wave-Kondortech, São Carlos, Brazil)	660 nm	CW	Fiber	Contact	30 mW	NS	1 cm^2^, 18 points	NS	NS	Twice weekly	2 J/cm^2^	NS	NS
Lima et al. [11]	Diode laser (Laser Unit KM 3000; DMC, São Carlos, SP, Brazil)	830 nm	CW	Fiber	NS	Nominal: 60 mW Effective: 15 mW	75 mW/cm^2^	0.2 cm^2^	160 s 12 sites	NS	Daily session (Monday–Friday) since the first day up to the end of RT	12 J/cm^2^	28.8 J/session	NS
Carvalho et al. [12]	InGaAlP diode laser (Twin laser MMOptics, MMOptics Ltda., São Carlos, São Paulo, Brazil)	660 nm	CW	Fiber	NS	G1: 15 mW G2: 5 mW	G1: 375 mW/cm^2^ G2: 125 mW/cm^2^	0.04 cm^2^	G1: 10 s G2: 10 s	NS	Daily session (Monday–Friday) since the first day up to the end of RT	G1: 3.8 J/cm^2^; G2: 1.3 J/cm^2^	NS	NS
Oton-Leite et al. [13]	InGaAlP diode laser (Thera Lase; DMC Equipments Ltda, Sao Carlos, Brazil)	685 nm	CW	Fiber	Contact	35 mW	NS	59 points	NS	NS	1/day for 5 consecutive days on 59 sites (a week before the beginning of RT/CT until the end of the treatment)	2 J/cm^2^	NS	NS
Gautam et al. [14]	Low level He–Ne laser (Technomed Electronics: Advanced Laser Therapy 1000)	632.8 nm	CW	Fiber	Non-contact	24 mW	24 mW/cm^2^	Spot size: 1 cm^2^	150–200 s 6 points	15–20 min/session 45 sessions	5 times/week prior to RT for 45 days	3 J/point	36–40 J/session	1,620–1,800 J/cm^2^
Gouvêa de Lima et al. [15]	GaAlAr diode laser (Twin Flex, MMOptics, São Carlos, Brazil)	660 nm	CW	Fiber	ns	10 mW	2.5 J/cm^2^	4 mm^2^	10 s per point	90 s	5 consecutive days (Monday–Friday) during all RT sessions	0.1 J	0.9 J	2.5 J/cm^2^
Gautam et al. [16]	He/Ne laser (Technomed Electronics, Advanced Laser Therapy 1000, Chennai, India)	632.8 nm	CW	Fiber	Non-contact (<1 cm)	24 mW	2.12 W/cm^2^	0.6 mm 6 sites	14.5 min	145 s	Daily for 6.5 weeks	NS	NS	3.5 J/cm^2^
Oton-Leite et al. [17]	InGaAlP diode laser (Thera Laser, DMC Equipments Ltd., Sao Carlos, Brazil)	685 nm	CW	Fiber	2 mm distant from the tissue	35 mW	NS	60 points 0.028 cm^2^	25 s/point	25 min/session	Start a week before the RT, daily for 5 consecutive days until the end of the RT	0.8 J per point	48 J/session	Min: 1,416 J Max: 1,888 J
Antunes et al. [18]	InGaAlP diode laser (DMC, São Carlos, São Paulo, Brazil)	660 nm	CW	Fiber	Contact	100 mW	NS	0.24 cm^2^ 9 areas	10 s	12 min	Once daily, 5 times/week	4 J/cm^2^	72 J/session	NS
Gautam et al. [19]	He-Ne laser (Technomed Electronics Advanced Laser Therapy 1000)	632.8 nm	NS	Fiber	NS	24 mW	24 mW/cm^2^	1 cm^2^	125 s on 6 sites	750 s/session	5 times/week	3 J/cm^2^	18 J/session	NS
Gobbo et al. [20]	Eltech.S.r.l. GaAlAs diode laser	970 nm	2 Hz, 50% duty cycle	Fiber	Distance	5,000 mW	NS	1 cm^2^ 9 sites	26 s/site on 9 sites	234 s	2/day for 4 consecutive days	NS	NS	NS
Oton-Leite et al. [21]	InGaAlP diode laser (Twin Flex Evolution, MMOptics Ltda, Sao Carlos, Brazil)	660 nm	CW	Fiber	Contact	25 mW	NS	61 points 0.04 cm^2^	10 s	610 s	3/week on alternate days for 7 weeks	6.2 J/cm^2^	15.13 J/session	317.69 J
Gautam et al. [22]	He/Ne laser (Technomed Electronics, Advanced Laser Therapy 1000, Chennai, India)	632.8 nm	CW	Fiber	Non-contact (<1 cm)	NS	0.024 mW/cm^2^	0.6 mm Spot size 1 cm^2^	125 s per 12 locations	NS	5 times a week	3 J/point	36 J/session	NS
Gonnelli et al. [23]	InGaAlP diode laser (Twin Laser—MMOptics® Ltda, São Carlos, SP, Brazil)	Extraoral application: 780 nm Intraoral application: 660 nm	CW	Fiber Array	Contact	Extraoral: 15 mW Intraoral: 40 mW	NS	0.04 cm^2^	Extraoral: 10 s per 16 points Intraoral: 10 s per 24 points	Extraoral: 160s Intraoral: 240s	3 times/week 21 sessions	Extraoral: 3.8 J/cm2 per point Intraoral: 10 J/cm^2^ per point	Extraoral: 2.432 J per session Intraoral: 9.6 J per session	3.8 J/cm^2^
Palma et al. [24]	InGaAlP diode laser device (Twin Flex III Evolution, MMOptics® Ltda, São Carlos, Brazil)	808 nm	CW	Fiber	Contact	30 mW	0.75 mW/cm^2^	Spot size 0.04 cm^2^	10 s per 22 points	3.6 min	24 sessions Twice/week for 3 months	0.3 J/point	6.6 J/session	7.5 J/cm^2^
Elgohary et al. [25]	Laser equipment (Electro Medical Supplies, Greenham Ltd., Wantage, Oxford- shire, UK)	950 nm	Pulsed 80%	Fiber	NS	15 mW	NS	NS	NS	6 min	5 times/week for 4 consecutive weeks	NS	4.3 J/cm^2^	86 J
González-Arriagada et al. [26]	Diode InGaAlP Photon Lase III (DMC Odontológica, São Carlos, Brazil)	660 nm	NS	Fiber	NS	100 mW	NS	NS	10 s 27 points	270 s	3 times/week since the first day up to the end of RT	60 J/cm^2^	NS	NS
Guedes et al. [27]	InGaArP Twin Flex Evolution (MM Optics Ltda, São Carlos, São Paulo, Brazil) and Laser Duo (MM Optics Ltda, São Carlos, São Paulo, Brazil)	660 nm	CW	Fiber	Contact	25 mW 100 mW	625 mW/cm^2^ 3,333 mW/cm^2^	4 mm^2^ 3 mm^2^	10 s/point 28 points	280 s	7 weeks	6.3 J/cm^2^ 33 J/cm2	7 J/session 28 J/session	NS
Legouté et al. [28]	He-Ne laser HETSCHL®	658 nm	Pulsed (50 Hz)	Fiber	0.5 mm	100 mW	100 mW/cm^2^	1 cm^2^ per application	40 s/cm^2^	NS	1 session/day, 5 sessions/week from day of OM grade II till the resolution OM	4 J	NS	4 J/cm^2^
Rezk-Allah et al. [29]	Infrared GaAs laser Phyaction CL- 904 device (Uniphy technology, Belgium)	904 nm	Pulse (200 ns)	Fiber	NS	25 W	NS	NS	60 s	NS	6 days/week from the start of OM till the end of CT	1 J/cm^2^	NS	NS
Bourbonne et al. [30]	Laser Heltschl FL 3500 ME-TL 10 000 SK (Schlüßlberg, Austria)	660 nm 658 nm	CW	Array	External: non-contact (1 cm) Intraoral: ns	External: 350 mW Intraoral: 100 mW	ns	External: 2 points Intraoral: 1 point	External: 4 mins Intraoral: ns	External: 8 mins Intraoral: ns	3 times/week for 7 weeks	6 J/cm^2^	12 J/cm^2^ 6 J/cm^2^	252 J 126 J
Morais et al. [31]	InGaAIP laser (Twin Flex Evolution, MM Optics Ltd., São Paulo, Brazil)	660 nm	CW	Fiber	1 cm distance	25 mW	NS	62 spots/0.04 mm^2^	10 s/site	620 s/session	5 days/week	6.2 J/cm^2^	14.88 J/day	446.4 J
Dantas et al. [32]	InGaAlP diode, Twin Flex (MM Optics, São Carlos, Brazil)	660 nm	CW	Fiber	Distance	86.7 mW	690 mW/cm^2^	0.1256 cm^2^	3 s	84 s (28 areas)	3x/week (Monday, Wednesday, Friday) from first day of RT	2 J/cm^2^	56 J/session	NS
Park et al. [33]	HEALITE II® 1800 light-emitting diodes (Lutronic Corp., Boston, MA, USA and Goyang, South Korea)	830 ± 7 nm	ns	Fiber	Contact	ns	100 mW/cm^2^	ns	660 s	660 s	3 times/week from the first week of RT. In average, 14.97 times (range from 12 to 18 times)	60 J/cm^2^	NS	37.80 J
De Carvalho et al. [34]	InGaAlP diode laser (Twin laser MMOptics, MMOptics Ltda., São Carlos, São Paulo, Brazil)	660 nm	CW	Fiber	Contact	15 mW 25 mW	375 mW/cm^2^ 625 mW/cm^2^	0.4 cm^2^/point 40 points	10 s	400 s	5 times/week from the first day until the end of RT	3.8 J/cm^2^ 6.3 J/cm^2^	152 J/cm^2^ 252 J/cm^2^	4,560 J/cm^2^ 7,560 J/cm^2^
Ribeiro et al. [35]	Flash AsGaAl Laser III (DMC, São Paulo Brazil)	808 nm	CW	Fiber	Distance	Intraoral: 15 mW External: 30 mW	NS	Intraoral: 0.028 cm^2^ 21 points Extraoral: 0.028 cm^2^ 18 points	10 s/point	Intraoral: 210s Extraoral: 180s	3 times/week on alternate days throughout the RT	Intraoral: 12 J/cm^2^ Extraoral: 7.5 J/cm2	50.4 J	NS
de Pauli Paglioni et al. [36]	Diode laser (Twin Flex, MM Optics Equipment, São Paulo, Brazil)	660 nm	CW	Fiber	Contact	40 mW	1,000 mW/cm^2^	0.04 cm2	Preventive:10 s Treatment:60 s	ns	Daily for 5 consecutive days/week from day 1 until the end of RT	Preventive: 10 J/cm^2^ Treatment: 60 J/cm^2^	600 J/cm^2^ for 10 sites	ns
Martins et al. [37]	Diode laser (Twin Flex Evolution, MM Optics Equipment, São Paulo, Brazil)	660 nm	CW	Fiber	Contact	25 mW	625 mW/cm^2^	0.04 cm^2^ 61 points	10 s	610 s	5 times/week from the first RT dose until the last one	0.25 J	6.2J/cm^2^	NS
Robijns et al. [38]	MLS® M6 diode laser (ASA Srl, Vicenza, Italy)	808 nm 905 nm	Continuous + pulsed wave mode 90 KHz	Array	5 cm above	1,100–2,500 mW (mean 3,300 mW)	168 mW/cm^2^	2 cm aperture, 3.14 cm^2^ at target	NS	300–600 s	Biweekly for 7 weeks	4 J/cm^2^	NS	NS
Bensadoun et al. [39]	Caremin 650	650 nm	CW	Array	Contact	NS	28 mW/cm^2^ for oral pads 21 mW/cm^2^ for derma pads	NS	NS	Prophylactic: 1 min 47 s (oral pads), 2 min 23 s (derma pads) Curative: 3 min 34 s (oral pads), 4 min 46 s (derma pads)	At least 3 sessions/week (5 sessions/week recommended) immediately before or after RT	NS	NS	3J/cm^2^ (prophylactic) 6 J/cm^2^ (curative)

### PBM for Oral Mucositis

The results for OM management were consistent, and guidelines for both prevention and treatment could be outlined in the current narrative review (Supplementary Table 1). All Authors choose diode lasers, more often indium gallium aluminum phosphide (InGaAlP) diode laser, and Helium-Neon (He/Ne) laser. The most preferred wavelength was red (632–660 nm) for both prevention and treatment protocols in continuous wave (CW) mode using fiber in contact or reduced (<1 cm) distance. Power output reported varied (5–5,000 mW), but most papers did not discriminate between nominal and effective, resulting in overestimated values, especially in non-contact protocols. A suggestion could be between 10 and 100 mW effective power. While some Authors mention irradiance per treatment point, others suggest a defocused beam ranging between 0.024 and 150 mW/cm^2^. As per the new PBM dosing, the most effective preventive protocol would use a total dose of 1.2 Einstein (photon fluence at 650 nm = 5.7 p.J/cm^2^). The data suggests successive intraoral applications on single spots on the oral cavity, rather than a scanning motion over the entire mucosal surface, may offer the most predictable outcomes. Also, the time of application was very variable, ranging from sessions of 270 s to 25 min. A minimum of 30 s per point with three (up to 5) sessions a week is recommended in preventive and treatment protocols. Overall, preventive protocols need more repetitions per week than treatment protocols.

### PBM for Xerostomia

All authors employed diode lasers, specifically indium gallium aluminum phosphide (InGaAlP) or Gallium Aluminum Arsenide (GaAlAs), preferring low power protocols (Supplementary Table 2). Both visible red (650–660 nm) and infrared (780–808 nm) wavelengths were used in CW mode. In two cases, the application was both intraoral and extraoral. Output power varied consistently, ranging from 10 to 100 mW for intraoral to 15–30 mW for extraoral applications. Also, time per site reported significantly gone from 3 to 400 s. Fluence went between 2 and 60 J/cm^2^, equating to 3.8–114 p.J/cm^2^ (photon fluence at 650 nm) or 0.8–25 Einstein. Sessions should be repeated at least twice a week but would be best effective if performed each day of RT (5-day per week), both in preventive and therapeutic protocols.

### PBM for Radiodermatitis

Among the four papers dealing with PBM for dermatitis management, two proposed a red wavelength, while the other used infrared (Supplementary Table 3). All Authors employed very heterogeneous diode devices (e.g., He/Ne, InGaAlP). Only Robjins et al. studied dermatitis specifically, while other authors did not distinguish between prevention or treatment of specific side effects [38]. Outputs varied between 100 and 2,500 mW and irradiance between 100 and 168 mW/cm^2^ when mentioned. The fluence varied between 2 and 60 J/cm^2^, equating to 3.8 to 114 p.J/cm^2^ (photon fluence at 650 nm) or 0.8 to 25 Einstein. Treatment time per session varied from 270 to 720 s while repetitions varied between 2 and 5 times a week for the whole course of RT. Although the publications on this topic are scarce and heterogeneous, there is a feeling toward the appropriateness of 2 or 3-weekly applications instead of daily sessions, preferring a preventive or combined strategy rather than just using PBM in a curative way. DeLand et al. reported that LED treatments immediately after RT reduces dermatitis incidence in breast cancer patients. These findings may inspire a protocol for HNC subjects. Despite the variability of the parameters, a general recommendation can be hypothesized [40].

### PBM for Pain and Trismus

PBM treatments for the management of pain and trismus induced by RT were assessed by two papers (Supplementary Table 4) [26]. While both protocols were focused on treatment, and the parameters were too heterogeneous for comparison, such as wavelength (660 red vs. 950 infrared), output powers (100 vs. 15 mW), and fluences (60 vs. 7.6 J/cm^2^ per session). Further, Elgohary et al. compared various techniques, including PBM, that were not the study's primary objective [25]. Based on our clinical experience, we recommend using a combination of 660 and 810 nm PBM devices, both intraoral and extraoral, at 50 mW/cm^2^ for 30 s per site, treating multiple areas in a scanning motion for a total fluence of 6 J/cm^2^ which equates to 9 p.J/cm^2^ at 810 nm or 2 Einstein. Treatments should be repeated up to 3 times per week for at least 3–4 weeks.

## Discussion

The present review offers an overview of the literature on PBM therapy in HNC patients with RT-related side effects, specifically OM, xerostomia, dermatitis, pain, and trismus. The most studied side effect of cancer treatments remains OM [41]. Literature has increased substantially, outlining preventive, therapeutic, or combined protocols [42]. The results section of our literature review has provided reliable suggestions for creating an effective protocol. PBM biological responses depend on the treatment parameters, delivery protocols, and redox state of the cells. It is well-established that PBM dosing is biphasic and relies on the underlying pathology and patient-associated factors that may affect individual outcomes. Further, inappropriate dosing may result in poor or adverse therapeutic effects. The PBM dose window is defined by correct treatment timing, the number of repetitions, and specific adaptation of protocols for each indication [43].

In general, PBM was noted to be effective in both the prevention and treatment of OM [27, 32]. It is almost universally accepted that the primary goal of treatment is reducing pain and improving QoL; most studies confirmed this regardless of the protocol. Even the low PBM efficacy papers noted reduced severity of OM grades (scores 3 and 4 according to the World Health Organization scale) and fewer treatment interruptions during RT. Most of the papers included in our systematic review used CW protocols. This contrasts with prior reports that pulsed, low-frequency (<100 Hz) may be superior for wound healing or the damage prevention. Moreover, while most studies used intraoral PBM treatments, there is evidence for extra-orally administered PBM that appears to be more effective for managing of OM of the buccal mucosa, vestibule, and inner lips when combined with an intraoral approach [44, 45].

The PBM studies on salivary glands after RT employed combined external and intraoral applications with both infrared and visible red wavelengths [17, 23]. There appears to be a dose-effect relationship for PBM on reduction of hyposalivation after RT, especially after 15 sessions with red or combined red and infra-red wavelengths [46]. For example, Ribeiro et al. conducted a cross-sectional study with a quantitative approach applying extraoral infrared PBM during the whole course of RT. They demonstrated unchanged unstimulated salivary flow during RT but decreased saliva quantity 1 month after the end of cancer treatment. Despite not corroborating the role of PBM in modulating hyposalivation and salivary gland damage, a concomitant intraoral, lower dose protocol was used for OM that was not the main objective of the study confounding the interpretations of their results [35]. Interestingly, the control of hyposalivation induced by RT seems to be positively affected by PBM treatment strategies [47]. On the contrary, the effect was not marked in preventive protocols. Three studies did not evidence a beneficial impact of PBM in reducing salivary flow connected to RT or combined CT/RT [15, 32, 35]. Note that only one of them is a randomized clinical trial and they all include a limited number of subjects. Moreover, there was no specific protocol for salivary complications that can be distinguished from other side effects, such as OM.

All the publications included in this narrative review suggest that PBM is a safe and valuable strategy for cutaneous complications in the HNR. Encouraging results were noted for PBM management or prevention of radiodermatitis. Many papers have been published regarding radiodermatitis in other body districts, breast *in primis*. However, little has been investigated in the cervical and facial sites, although it is associated with significant pain, disfigurement, risk of RT interruption, and poor cancer prognosis [38]. For cutaneous areas other than the HNR, the literature suggests that preventive PBM application, starting concomitantly or even before RT or combined CT/RT, may not only mitigate the severity of dermatitis but also positively impact the onset and severity of late complications, *via* the mechanisms of tissue repair and regeneration. For example, a study on pigs suggested that combined wavelengths positively influence the development of late radiation damage to the skin. This indicates that this approach may also be applied in the HNR [48]. The fact that all the included publications were very recent (2018–2022) indicates increased interest and recognition of the efficacy of this treatment, together with its proven safety, suggesting that a universal protocol may be feasible shortly.

Specific interest has emerged in this review in trismus management, which is not corroborated by previous literature work. HNC patients are often subdued to destructive surgery, which provokes muscle spasms and reduced mouth opening. The evidence that PBM reduces fibrosis and promotes muscle regeneration could be the primary rationale for the clinical benefit looked for by the Authors, even if it is evident that this topic needs further clinical research [45].

In summary, the available evidence shows that PBM was satisfactory in managing complications related to cancer therapies, both in the prevention of onset and in the reduction of severity and duration, especially for OM. Objective and subjective parameters were studied with comparable rates of success, and the favorable implications on QoL outcomes and wellbeing accounted for most of the positive results expressed by the authors [37]. PBM generates beneficial effects, including reducing of inflammation and pain [49], promoting tissue repair, reducing fibrosis, and favoring nerve regeneration. Therefore, it is clear why studies on PBM application cover a vast range of acute and chronic cancer-related complications in HNC patients.

Moreover, there is growing evidence that PBM is cost-effective both in preventing and treating cancer treatment-related toxicities, such as OM and breast cancer-related lymphedema. This scenario may provide a wider acceptance of PBM at cancer treatment centers, especially if fomented by additional clinical studies to validate cost-effectiveness for preventing and managing cancer treatment-related toxicities other than OM [50].

PBM dosimetry has raised significant interest in recent years, primarily due to its efficacy in a broad range of clinical applications, regardless of the underlying pathology and varying protocols. But since Mester's first description of its benefits, PBM has been used rather empirically as a magic wand, without actual knowledge of photobiological, molecular, and intercellular mechanisms of laser-tissue interaction that cannot be ignored [51]. The absence of clear guides for standardizing protocols description and data presentation remains an issue that can limit comparison among studies and the creation of coherent clinical practice guidelines. Inconsistencies in clinical outcomes are mainly due to problems in reporting PBM dosing and delivery. For the latter, using “*treatment surface irradiance*” rather than laser irradiance alone is expected to reduce confusion about power output, spot size, and distance, especially when using contact and defocused (distant) PBM treatments [24]. This should assist in significantly improving dose reproducibility. The availability of large arrays has encouraged defocused, large treatment areas that reduce treatment time and thermal damage in tissues. Eventually, disease-focused protocols could be created as specific wavelengths target biological chromophores at varying penetration depths and evoke discrete biological responses. Universal protocols may seem convenient and somewhat effective, they are likely to generate inconsistent or irreproducible results [52].

Even in the case of different protocols applied to the same condition, the evoked PBM responses may vary. The absorption of light by a chromophore depends on the affinity with the used wavelength. Even if the wavelength falls within the correct absorption spectrum, low doses of energy are insufficient to start the biological effect, and excessive dosages can result in inhibitory. Moreover, therapeutic responses are restricted to a limited therapeutic dose window termed the Arndt Schultz curve [53]. Recent papers emerged in the literature regarding the possibility of enabling comparisons between protocols, creating a system of “dosing consistency,” which is effective with multiple combined wavelengths. Young et al. suggested using the terms photonic fluence (p.J/cm^2^) and “Einstein” (photonic fluence at 810 nm as a reference wavelength) [51]. This enables easy, universal interoperability between dose recommendations with different wavelengths. This novel dose system has been recently applied to the dosing recommendations by the World Association for Photobiomodulation Therapy (WALT) to increase practical implementation irrespective of individual wavelengths or devices that are available globally while preventing overdosing and enabling dose combination with various wavelengths [51].

The similarities of the pathophysiology in different complications and the fact that the same patients may suffer from more than one side effect represent a clear clinical challenge. Moreover, based on the logical extension of acute complications as precursors for chronic ones, preventive (“pre-conditioning”) PBM protocols could effectively reduce early and late complications [54]. PBM should be applied using the optimal parameters based on the biological target, device parameters, and delivery technique. Therefore, it is rational to posit that optimal protocols could maximize clinical efficacy, creating a reproducible, and consistent treatment irrespective of the device being used. This work attempts to outlining some of these parameters to pave the way for universal PBM protocols.

## Conclusion

PBM seems to be an efficacious intervention for several complications of cancer therapy. Robust evidence of the clinical benefit elicited by the correct biological and molecular patterns of light stimulation exists. There is a strong perception that multiple protocols may be applied to similar conditions but to maximize the effect on specific tissue targets, there is an urgent need for standardization and reproducibility of dosages. The increasing number of papers regarding the management of HNC complications *via* PBM witnesses a strong interest in the field. The very recent publications proposing dosage standardization indicate we are moving in the right direction.

## Author Contributions

GO and MG contributed to conception and design of the study. MG, EM, PA, R-JB, AS-S, LG, and GO performed the articles screening and data collection. MG wrote the first draft of the manuscript. EM, PA, R-JB, AS-S, LG, and GO wrote sections of the manuscript. All authors contributed to manuscript revision, read, and approved the submitted version.

## Funding

The authors gratefully acknowledge the support of Eltech K-Laser Company for the publication financial support. Eltech K-Laser Company was not involved in the study design, collection, analysis, interpretation of data, the writing of this article or the decision to submit it for publication.

## Conflict of Interest

The authors declare that the research was conducted in the absence of any commercial or financial relationships that could be construed as a potential conflict of interest.

## Publisher's Note

All claims expressed in this article are solely those of the authors and do not necessarily represent those of their affiliated organizations, or those of the publisher, the editors and the reviewers. Any product that may be evaluated in this article, or claim that may be made by its manufacturer, is not guaranteed or endorsed by the publisher.
