# Supplementary material for: Quality Assessment of PBM Protocols for Oral Complications in Head and Neck Cancer Patients: Part 1
Source: Front Oral Health. 2022 Jul 7;3:945718. doi: 10.3389/froh.2022.945718 (PMC9300948; doi:10.3389/froh.2022.945718)
Supplement: Supplementary file 1 [file Table_1.docx]

**Supplementary table 1. Photobiomodulation in the management of oral mucositis.**

| **Paper** | **Type**  **brand** | **Wavelength** | **Mode (CW/Pulse)** | **Format (Fiber, array)** | **Contact or Distance** | **Power output (mW)** | **Irradiance (mW/cm2)** | **Spots/**  **area** | **Time/**  **site** | **Time/**  **session** | **Repetitions** | **Fluence/**  **site** | **Fluence/**  **session** | **Total Fluence** |
| --- | --- | --- | --- | --- | --- | --- | --- | --- | --- | --- | --- | --- | --- | --- |
| **Bensadoun RJ, 1999 ^5^** | Low-energy  He-Ne laser (Fradama Geneva, Switzerland) | 632.8 nm | CW | Fiber | 0.5 mm | 60 mW | ns | 1 cm2/point 9 points | 33 s per spot (Nice and Marseilles)  80 s per spot (Reims) | 5 min/session (Nice and Marseilles)  12 min/session (Reims) | 5 days/week (Monday to Friday) for 7 consecutive weeks | 2 J/cm2 | 18 J | 3 J/cm2 |
| **Arun Maiya G, 2006 ^6^** | He-Ne laser (Electro care Ltd. Laser 2001, India) | 632.8 nm | ns | Fiber | ns | 10 mW | ns | ns | ns | 3 min/session | 5 days/week | 1.8 J/cm2 | ns | ns |
| **Lopes CO, 2006 ^7^** | InGaAlP laser | 685 nm | ns | Fiber | Contact | 50 mW (nominal power) 35 mW (real power) | Diameter of 400 µm | 0.028 cm2  19 points | ns | 58 s | 10 days | 2 J/point | ns | 70 J/cm2 |
| **Arora H, 2008 ^8^** | He-Ne laser (Electro Care Ltd, Laser 2001, Chennai, India) | 632.8 nm | Pulse (10 Hz) for 8 days, then CW for 25 days | Scanner for 8 days, fiber for the following 25 days | Distance | 10 mW | ns | ns | 5 min/site on 6 sites | First 8 days: 5 mins supine position, following 25 days: 30 mins | 33 sessions | 1.8 J/cm2 | ns | ns |
| **Zanin T, 2010 ^10^** | AlGaInP diode laser (Bio Wave-Kondortech, São Carlos, Brazil) | 660 nm | CW | Fiber | Contact | 30 mW | ns | 1 cm2,  18 points | ns | ns | Twice weekly | 2 J/cm2 | ns | ns |
| **Lima AG, 2010 ^11^** | Diode laser (Laser Unit KM 3000; DMC, São Carlos, SP, Brazil) | 830 nm | CW | Fiber | ns | Nominal: 60 mW Effective: 15 mW | 75 mW/cm2 | 0.2 cm2 | 160 s  12 sites | ns | Daily session (Monday to Friday)  since the first day up to the end of RT | 12 J/cm2 | 28.8 J/session | ns |
| **Carvalho PA, 2011 ^12^** | InGaAlP diode laser (Twin laser MMOptics, MMOptics Ltda., São Carlos, São Paulo, Brazil) | 660 nm | CW | Fiber | ns | G1: 15 mW  G2: 5 mW | G1: 375 mW/cm2  G2: 125 mW/cm2 | 0.04 cm2 | G1: 10 s  G2: 10 s | ns | Daily session (Monday to Friday)  since the first day up to the end of RT | G1: 3.8 J/cm2; G2: 1.3 J/cm2 | ns | ns |
| **Oton-Leite AF, 2012 ^13^** | InGaAlP diode laser (Thera Lase; DMC Equipments Ltda, Sao Carlos, Brazil) | 685 nm | CW | Fiber | Contact | 35 mW | ns | 59 points | ns | ns | 1/day for 5 consecutive days on 59 sites (a week before the beginning of RT/CT until the end of the treatment) | 2 J/cm2 | ns | ns |
| **Gautam AP, 2012 ^14^** | Low level He–Ne laser (Technomed Electronics: Advanced Laser Therapy 1000) | 632.8 nm | CW | Fiber | Non-contact | 24 mW | 24 mW/cm2 | Spot size:  1 cm2 | 150-200 s  6 points | 15-20 min/session  45 sessions | 5 times/week  prior to RT for 45 days | 3 J/point | 36-40 J/session | 1620-1800 J/cm2 |
| **Gouvêa de Lima A,**  **2012 ^15^** | GaAlAr diode laser (Twin Flex, MMOptics, São Carlos, Brazil) | 660 nm | CW | Fiber | ns | 10 mW | 2.5 J/cm2 | 4 mm2 | 10s per point | 90s | 5 consecutive days (Monday to Friday) during all RT sessions | 0.1 J | 0.9 J | 2.5 J/cm2 |
| **Gautam AP, 2012 ^16^** | He/Ne laser (Technomed Electronics, Advanced Laser Therapy 1000, Chennai, India) | 632.8 nm | CW | Fiber | Non-contact (< 1cm) | 24 mW | 2.12 W/cm2 | 0.6 mm  6 sites | 14.5 mins | 145 s | Daily for 6.5 weeks | ns | ns | 3.5 J/cm2 |
| **Oton-Leite AF, 2013 ^17^** | InGaAlP diode laser (Thera Laser, DMC Equipments Ltd., Sao Carlos, Brazil) | 685 nm | CW | Fiber | 2 mm distant from the tissue | 35 mW | ns | 60 points  0.028 cm2 | 25 s/point | 25 min/session | Start a week before the RT, daily for 5 consecutive days until the end of the RT | 0.8 J per point | 48 J/session | Min: 1416 J Max: 1888 J |
| **Antunes HS, 2013 ^18^** | InGaAlP diode laser (DMC, São Carlos, São Paulo, Brazil) | 660 nm | CW | Fiber | Contact | 100 mW | ns | 0.24 cm2  9 areas | 10 s | 12 min | Once daily, 5 times/week | 4 J/cm2 | 72 J/session | ns |
| **Gautam AP, 2013 ^19^** | He-Ne laser (Technomed Electronics Advanced Laser Therapy 1000) | 632.8 nm | ns | Fiber | ns | 24 mW | 24 mW/cm2 | 1 cm2 | 125 s  on 6 sites | 750 s/session | 5 times/week | 3 J/cm2 | 18 J/session | ns |
| **Gobbo M, 2014 ^20^** | Eltech.S.r.l.  GaAlAs diode laser | 970 nm | 2 Hz, 50% duty cycle | Fiber | Distance | 5000 mW | ns | 1 cm2  9 sites | 26 s/site  on 9 sites | 234 s | 2/day for 4 consecutive days | ns | ns | ns |
| **Oton-Leite AF, 2015 ^21^** | InGaAlP diode laser (Twin Flex Evolution, MMOptics Ltda, Sao Carlos, Brazil) | 660 nm | CW | Fiber | Contact | 25 mW | ns | 61 points  0.04 cm2 | 10 s | 610 s | 3/week on alternate days for 7 weeks | 6.2 J/cm2 | 15.13 J/session | 317.69 J |
| **Gautam AP, 2015 ^22^** | He/Ne laser (Technomed Electronics, Advanced Laser Therapy 1000, Chennai, India) | 632.8 nm | CW | Fiber | Non-contact (< 1cm) | ns | 0.024 mW/cm2 | 0.6 mm  spot size  1 cm2 | 125 s per 12 locations | ns | 5 times a week | 3 J/point | 36 J/session | ns |
| **Gonnelli FAS, 2016 ^23^** | InGaAlP diode laser (Twin Laser - MMOptics® Ltda, São Carlos, SP, Brazil) | Extraoral application: 780 nm  Intraoral application: 660 nm | CW | Fiber      Array | Contact | Extraoral: 15 mW    Intraoral: 40 mW | ns | 0.04 cm2 | Extraoral: 10 s per 16 points  Intraoral: 10 s per 24 points | Extraoral: 160s      Intraoral: 240s | 3 times/week  21 sessions | Extraoral: 3.8 J/cm2 per point  Intraoral: 10 J/cm2 per point | Extraoral: 2.432 J per session  Intraoral: 9.6 J per session | 3.8 J/cm2 |
| **Palma LF, 2017 ^24^** | InGaAlP diode laser device (Twin Flex III Evolution, MMOptics® Ltda, São Carlos, Brazil) | 808 nm | CW | Fiber | Contact | 30 mW | 0.75 mW/cm2 | Spot size  0.04 cm2 | 10 seconds per 22 points | 3.6 min | 24 sessions  twice/week  for 3 months | 0.3 J/point | 6.6 J/session | 7.5 J/cm2 |
| **González-Arriagada WA, 2018 ^26^** | Diode InGaAlP Photon Lase III (DMC Odontológica, São Carlos, Brazil) | 660 nm | ns | Fiber | ns | 100 mW | ns | ns | 10 s  27 points | 270 s | 3 times/week since the first day up to the end of RT | 60 J/cm2 | ns | ns |
| **Guedes CDCFV, 2018 ^27^** | InGaArP Twin Flex Evolution (MM Optics Ltda, São Carlos, São Paulo, Brazil) and Laser Duo (MM Optics Ltda, São Carlos, São Paulo, Brazil) | 660 nm | CW | Fiber | Contact | 25 mW  100 mW | 625 mW/cm2 3,333 mW/cm2 | 4 mm2  3 mm2 | 10 s/point  28 points | 280 s | 7 weeks | 6.3 J/cm2  33 J/cm2 | 7 J/session  28 J/session | ns |
| **Legouté F, 2019 ^28^** | He-Ne laser HETSCHL® | 658 nm | Pulsed (50 Hz) | Fiber | 0.5 mm | 100 mW | 100  mW/cm2 | 1 cm2 per application | 40 s/cm2 | ns | 1 session/day, 5 sessions/week from day of OM grade II till the resolution OM | 4 J | ns | 4 J/cm2 |
| **Rezk-Allah SS, 2019 ^29^** | Infrared GaAs laser Phyaction CL- 904 device (Uniphy technology, Belgium) | 904 nm | Pulse  (200 ns) | Fiber | ns | 25 W | ns | ns | 60 s | ns | 6 days/week from the start of OM till the end of CT | 1 J/cm2 | ns | ns |
| **Bourbonne V, 2019 ^30^** | Laser Heltschl FL 3500  ME-TL 10 000 SK (Schlüßlberg, Austria) | 660 nm  658 nm | CW | Array | External: non-contact (1 cm)  Intraoral: ns | External: 350mW  Intraoral: 100mW | ns | External:  2 points  Intraoral:  1 point | External:  4 mins  Intraoral: ns | External:  8 mins  Intraoral: ns | 3 times/week for 7 weeks | 6 J/cm2 | 12 J/cm2  6 J/cm2 | 252 J  126 J |
| **Morais MO, 2020 ^31^** | InGaAIP laser (Twin Flex Evolution, MMOptics Ltd., São Paulo, Brazil) | 660 nm | CW | Fiber | 1 cm distance | 25 mW | ns | 62 spots/  0.04 mm2 | 10 s/site | 620 s/session | 5 days/week | 6.2 J/cm2 | 14.88 J/day | 446.4 J |
| **Dantas JBL, 2020 ^32^** | InGaAlP diode, Twin Flex (MM Optics, São Carlos, Brazil) | 660 nm | CW | Fiber | Distance | 86.7 mW | 690 mW/cm2 | 0.1256 cm2 | 3 s | 84 s  (28 areas) | 3x/week (Monday, Wednesday, Friday) from first day of RT | 2 J/cm2 | 56 J/session | ns |
| **De Carvalho PAG, 2020 ^34^** | InGaAlP diode laser (Twin laser MMOptics, MMOptics Ltda., São Carlos, São Paulo, Brazil) | 660 nm | CW | Fiber | Contact | 15 mW    25 mW | 375 mW/cm2  625 mW/cm2 | 0.4 cm2/point  40 points | 10 sec | 400 sec | 5 times/week from the first day until the end of RT | 3.8 J/cm2  6.3 J/cm2 | 152 J/cm2  252 J/cm2 | 4560 J/cm2  7560 J/cm2 |
| **de Pauli Paglioni M, 2021 ^36^** | Diode laser (Twin Flex, MMOptics Equipment, São Paulo, Brazil) | 660 nm | CW | Fiber | Contact | 40 mW | 1000 mW/cm2 | 0.04 cm2 | Preventive:10 s  Treatment:60 s | ns | Daily for 5 consecutive days/week from day 1 until the end of RT | Preventive: 10 J/cm2  Treatment: 60 J/cm2 | 600 J/cm2 for 10 sites | ns |
| **Martins AFL, 2021 ^37^** | Diode laser (Twin Flex Evolution, MMOptics Equipment, São Paulo, Brazil) | 660 nm | CW | Fiber | Contact | 25 mW | 625 mW/cm2 | 0.04 cm2  61 points | 10 s | 610 s | 5 times/week from the first RT dose until the last one | 0.25 J | 6.2J/cm2 | ns |
