# Supplementary material for: Quality Assessment of PBM Protocols for Oral Complications in Head and Neck Cancer Patients: Part 1
Source: Front Oral Health. 2022 Jul 7;3:945718. doi: 10.3389/froh.2022.945718 (PMC9300948; doi:10.3389/froh.2022.945718)
Supplement: Supplementary file 2 [file Table_2.docx]

**Supplementary table 2. Photobiomodulation in the management of xerostomia.**

| **Paper** | **Type**  **brand** | **Wavelength** | **Mode (CW/Pulse)** | **Format (Fiber, array)** | **Contact or Distance** | **Power output (mW)** | **Irradiance (mW/cm2)** | **Spots/**  **area** | **Time/**  **site** | **Time/**  **session** | **Repetitions** | **Fluence/**  **site** | **Fluence/**  **session** | **Total Fluence** |
| --- | --- | --- | --- | --- | --- | --- | --- | --- | --- | --- | --- | --- | --- | --- |
| **Lopes CO, 2006 ^7^** | InGaAlP laser | 685 nm | ns | Fiber | Contact | 50 mW (nominal power) 35 mW (real power) | Diameter of 400 µm | 0.028 cm2  19 points | ns | 58 s | 10 days | 2 J/point | ns | 70 J/cm2 |
| **Simões A, 2009 ^9^** | Low Power Laser: InGaAlP diode laser (Twin Flex III Evolution, MMOptics® Ltda, São Carlos, Brazil)  Combined Low/High Power Lasers: GaAlAs diode laser (Soft Lase, Zap Laser Ltd, Pleasant Hill, CA) | Low Power Laser: 660 nm  Combined Low/High Power Lasers: 808 nm | CW | Fiber | Non-contact  1 cm from the lesion | 40 mW | Low Power Laser: 40 mW/cm2  Combined Low/High Power Lasers: 1 W/cm2 | 0.036 cm2 | Low Power Laser: 6 s per 62 points  Combined Low/High Power Lasers: 10 s on ulcers | Low Power Laser: 372 s  Combined Low/High Power Lasers: ns | 1-3 times/week for 8 months | Low Power Laser: 0.24 J/point | Low Power Laser: 6 J/cm2 | Low Power Laser: 3.8 J/cm2 |
| **Gouvêa de Lima A,**  **2012 ^15^** | GaAlAr diode laser (Twin Flex, MMOptics, São Carlos, Brazil) | 660 nm | CW | Fiber | ns | 10 mW | 2.5 J/cm2 | 4 mm2 | 10s per point | 90s | 5 consecutive days (Monday to Friday) during all RT sessions | 0.1 J | 0.9 J | 2.5 J/cm2 |
| **Gonnelli FAS, 2016 ^23^** | InGaAlP diode laser (Twin Laser - MMOptics® Ltda, São Carlos, SP, Brazil) | Extraoral application: 780 nm  Intraoral application: 660 nm | CW | Fiber     Array | Contact | Extraoral: 15 mW    Intraoral: 40 mW | ns | 0.04 cm2 | Extraoral: 10 s per 16 points  Intraoral: 10 s per 24 points | Extraoral: 160s     Intraoral: 240s | 3 times/week  21 sessions | Extraoral: 3.8 J/cm2 per point  Intraoral: 10 J/cm2 per point | Extraoral: 2.432 J per session  Intraoral: 9.6 J per session | 3.8 J/cm2 |
| **Palma LF, 2017 ^24^** | InGaAlP diode laser device (Twin Flex III Evolution, MMOptics® Ltda, São Carlos, Brazil) | 808 nm | CW | Fiber | Contact | 30 mW | 0.75 mW/cm2 | Spot size  0.04 cm2 | 10 seconds per 22 points | 3.6 min | 24 sessions  twice/week  for 3 months | 0.3 J/point | 6.6 J/session | 7.5 J/cm2 |
| **González-Arriagada WA, 2018 ^26^** | Diode InGaAlP Photon Lase III (DMC Odontológica, São Carlos, Brazil) | 660 nm | ns | Fiber | ns | 100 mW | ns | ns | 10 s  27 points | 270 s | 3 times/week since the first day up to the end of RT | 60 J/cm2 | ns | ns |
| **Morais MO, 2020 ^31^** | InGaAIP laser (Twin Flex Evolution, MMOptics Ltd., São Paulo, Brazil) | 660 nm | CW | Fiber | 1 cm distance | 25 mW | ns | 62 spots/  0.04 mm2 | 10 s/site | 620 s/session | 5 days/week | 6.2 J/cm2 | 14.88 J/day | 446.4 J |
| **Dantas JBL, 2020 ^32^** | InGaAlP diode, Twin Flex⃝R (MM Optics, São Carlos, Brazil | 660 nm | CW | Fiber | Distance | 86.7 mW | 690 mW/cm2 | 0.1256 cm2 | 3 s | 84 s  (28 areas) | 3x/week (Monday, Wednesday, Friday) from first day of RT | 2 J/cm2 | 56 J/session | ns |
| **Ribeiro LN, 2021 ^35^** | Flash AsGaAl Laser III (DMC, São Paulo Brazil) | 808 nm | CW | Fiber | Distance | Intraoral: 15 mW  External:  30 mW | ns | Intraoral: 0.028 cm2  21 points  Extraoral:  0.028 cm2  18 points | 10 s/point | Intraoral: 210s  Extraoral: 180s | 3 times/week on alternate days throughout the RT | Intraoral:  12 J/cm2  Extraoral: 7.5 J/cm2 | 50.4 J | ns |
| **Bensadoun RJ, 2022 ^39^** | Caremin 650 | 650 nm | CW | Array | Contact | ns | 28 mW/cm2  for oral pads  21 mW/cm2  for derma pads | ns | ns | Prophylactic:  1 min 47 s (oral pads), 2 min 23 s (derma pads) Curative: 3 min 34 s (oral pads), 4 min 46 s (derma pads) | At least 3 sessions/week (5 sessions/week recommended) immediately before or after RT | ns | ns | 3J/cm2 (prophylactic)  6 J/cm2 (curative) |
