# Supplementary material for: Quality Assessment of PBM Protocols for Oral Complications in Head and Neck Cancer Patients: Part 1
Source: Front Oral Health. 2022 Jul 7;3:945718. doi: 10.3389/froh.2022.945718 (PMC9300948; doi:10.3389/froh.2022.945718)
Supplement: Supplementary file 3 [file Table_3.docx]

**Supplementary table 3. Photobiomodulation in the management of radio-induced dermatitis.**

| **Paper** | **Type**  **brand** | **Wavelength** | **Mode (CW/Pulse)** | **Format (Fiber, array)** | **Contact or Distance** | **Power output (mW)** | **Irradiance (mW/cm2)** | **Spots/**  **area** | **Time/**  **site** | **Time/**  **session** | **Repetitions** | **Fluence/**  **site** | **Fluence/**  **session** | **Total Fluence** |
| --- | --- | --- | --- | --- | --- | --- | --- | --- | --- | --- | --- | --- | --- | --- |
| **Bensadoun RJ, 1999 ^5^** | Low-energy  He-Ne laser (Fradama Geneva, Switzerland) | 632.8 nm | CW | Fiber | 0.5 mm | 60 mW | ns | 1 cm2/point 9 points | 33 s per spot (Nice and Marseilles)  80 s per spot (Reims) | 5 min/session (Nice and Marseilles)  12 min/session (Reims) | 5 days/week (Monday to Friday) for 7 consecutive weeks | 2 J/cm2 | 18 J | 3 J/cm2 |
| **González-Arriagada WA, 2018 ^26^** | Diode InGaAlP Photon Lase III (DMC Odontológica, São Carlos, Brazil) | 660 nm | ns | Fiber | ns | 100 mW | ns | ns | 10 s  27 points | 270 s | 3 times/week since the first day up to the end of RT | 60 J/cm2 | ns | ns |
| **Park JH, 2020 ^33^** | HEALITE II® 1800 light-emitting diodes (Lutronic Corp., Boston, MA, USA and Goyang, South Korea). | 830 nm ±  7 nm | ns | Fiber | Contact | ns | 100 mW/cm2 | ns | 660 s | 660 s | 3 times/week from the first week of RT. In average, 14.97 times (range from 12 to 18 times) | 60 J/cm2 | ns | 37.80 J |
| **Robijns J, 2021 ^38^** | MLS® M6 diode laser (ASA Srl, Vicenza, Italy) | 808 nm  905 nm | Continuous + pulsed wave mode 90KHz | Array | 5 cm above | 1100-2500 mW (mean 3300 mW) | 168 mW/cm2 | 2 cm aperture, 3.14 cm2 at target | ns | 300-600 s | Biweekly for 7 weeks | 4 J/cm2 | ns | ns |
