# Supplementary material for: Quality Assessment of PBM Protocols for Oral Complications in Head and Neck Cancer Patients: Part 1
Source: Front Oral Health. 2022 Jul 7;3:945718. doi: 10.3389/froh.2022.945718 (PMC9300948; doi:10.3389/froh.2022.945718)
Supplement: Supplementary file 4 [file Table_4.docx]

**Supplementary table 4. Photobiomodulation in the management of pain and trismus.**

| **Paper** | **Type**  **brand** | **Wavelength** | **Mode (CW/Pulse)** | **Format (Fiber, array)** | **Contact or Distance** | **Power output (mW)** | **Irradiance (mW/cm2)** | **Spots/**  **area** | **Time/**  **site** | **Time/**  **session** | **Repetitions** | **Fluence/**  **site** | **Fluence/**  **session** | **Total Fluence** |
| --- | --- | --- | --- | --- | --- | --- | --- | --- | --- | --- | --- | --- | --- | --- |
| **Elgohary HM, 2018 ^25^** | Laser equipment (Electro Medical Supplies, Greenham Ltd., Wantage, Oxford- shire, UK) | 950 nm | Pulsed 80% | Fiber | ns | 15 mW | ns | ns | ns | 6 mins | 5 times/week for 4 consecutive weeks | ns | 4.3 J/cm2 | 86 J |
| **González-Arriagada WA, 2018 ^26^** | Diode InGaAlP Photon Lase III (DMC Odontológica, São Carlos, Brazil) | 660 nm | ns | Fiber | ns | 100 mW | ns | ns | 10 s  27 points | 270 s | 3 times/week since the first day up to the end of RT | 60 J/cm2 | ns | ns |
